# Characterization of a malaria outbreak in Colombia in 2010

**DOI:** 10.1186/1475-2875-12-330

**Published:** 2013-09-17

**Authors:** Pablo Chaparro, Julio Padilla, Andrés F Vallejo, Sócrates Herrera

**Affiliations:** 1 National Institute of Health of Colombia, Bogotá, Colombia; 2 National University of Colombia, Bogotá, Colombia; 3 Ministry of Health and Social Protection of Colombia, Bogotá, Colombia; 4 Caucaseco Scientific Research Center, Cali, Colombia; 5 Latin American Center for Malaria Research, Cali, Colombia

**Keywords:** Colombia, Malaria surveillance, Epidemiology, *Plasmodium vivax*, *Plasmodium falciparum*

## Abstract

**Background:**

Although malaria has presented a significant reduction in morbidity and mortality worldwide during the last decade, it remains a serious global public health problem. In Colombia, during this period, many factors have contributed to sustained disease transmission, with significant fluctuations in an overall downward trend in the number of reported malaria cases. Despite its epidemiological importance, few studies have used surveillance data to describe the malaria situation in Colombia. This study aims to describe the characteristics of malaria cases reported during 2010 to the Public Health Surveillance System (SIVIGILA) of the National Institute of Health (INS) of Colombia.

**Methods:**

A descriptive study was conducted using malaria information from SIVIGILA 2010. Cases, frequencies, proportions, ratio and measures of central tendency and data dispersion were calculated. In addition, the annual parasite index (API) and the differences between the variables reported in 2009 and 2010 were estimated.

**Results:**

A total of 117,108 cases were recorded by SIVIGILA in 2010 for a national API of 10.5/1,000 habitants, with a greater number of cases occurring during the first half of the year. More than 90% of cases were reported in seven departments (=states): Antioquia: 46,476 (39.7%); Chocó: 22,493 (19.2%); Cordoba: 20,182 (17.2%); Valle: 6,360 (5.4%); Guaviare: 5,876 (5.0%); Nariño: 4,085 (3.5%); and Bolivar: 3,590 (3.1%). *Plasmodium vivax* represented ~71% of the cases; *Plasmodium falciparum* ~28%; and few infrequent cases caused by *Plasmodium malariae*.

**Conclusions:**

Overall, a greater incidence was found in men (65%) than in women (35%). Although about a third of cases occurred in children <15 years, most of these cases occurred in children >5 years of age. The ethnic distribution indicated that about 68% of the cases occurred in mestizos and whites, followed by 23% in Afro-descendants, and the remainder (9%) in indigenous communities. In over half of the cases, consultation occurred early, with 623 complicated and 23 fatal cases. However, the overall incidence increased, corresponding to an epidemic burst and indicating the need to strengthen prevention and control activities as well as surveillance to reduce the risk of outbreaks and the consequent economic and social impact.

## Background

Malaria is caused by one or more of the five species of *Plasmodium* that can infect humans (*Plasmodium vivax*, *Plasmodium falciparum*, *Plasmodium malariae*, *Plasmodium ovale*, *Plasmodium knowlesi*) after transmission by the bite of malarial female *Anopheles* mosquitoes. Although *P*. *falciparum* is highly prevalent worldwide, *P*. *vivax* has a wider distribution [1] and is the prevalent species outside the African continent. *Plasmodium ovale* and *P*. *malariae* are mainly transmitted in tropical areas of Africa and some islands of Southeast Asia [2], whereas *P*. *knowlesi* seems to be restricted to Southeast Asia [3]. Between 2005 and 2009, the estimated global number of malaria cases decreased from 237 million to 222 million, while the estimated number of malaria deaths during the same period decreased from >800,000 cases in 2005 to 691,000 cases in 2009 (~14%) [4].

In the American continent, malaria affects 23 countries, where about 20% of the population is at some risk of disease [5]. Brazil and Colombia are the major contributors of malaria cases in the region, accounting for 67% of the total malaria cases, followed by Haiti, Peru and Venezuela. Altogether the five countries accounted for 91% of the cases reported in the American continent in 2009 [5,6]. In general, *P*. *vivax* is responsible for most of the malaria cases of the continent (70%), which have been estimated to range between 1.9 million in 2005 and 1.1 million in 2009.

During the last decade several countries of the continent have presented a significant and stable decrease in malaria incidence: Peru and Ecuador in South America; and Guatemala, Honduras, El Salvador, Costa Rica, and Panama in Central America (decreased by >60% malaria transmission). However, in Colombia, during the same period, morbidity has remained high, with malaria cases fluctuating between 231,000 and 66,000 cases per year [5].

About 85% of the Colombian rural territory is at an altitude less than 1,600 m above sea level, and thus presents climatic, geographic and epidemiological conditions suitable for malaria transmission. It is estimated that approximately a quarter (~11 million people) of its current national population, estimated at 45 million people, live permanently at risk of infection [7]. This portion of the national population reside in areas where environmental factors such as temperature, humidity, precipitation, latitude and altitude, as well as social, cultural, economic and political factors compromise the effectiveness of prevention and control programs. Despite the relatively higher rate of malaria transmission in Colombia, it is still considered unstable with endemic-epidemic patterns and focal variables in different eco-epidemiological regions [8].

In general, during the last seven years, the Colombian National Malaria Control Programme (NMCP) has targeted its activities on reducing the interaction among vectors, parasites and human hosts through strategies such as case management, prevention and surveillance [9,10]. Malaria case management is focused on early detection, i.e., diagnosis of malaria cases and prompt and effective treatment of symptomatic patients. This is based on an extended diagnostic network with >1,700 laboratories and 1,195 microscopists where most cases are detected by passive surveillance using thick blood smears (TBS), although there is a growing use of rapid diagnostic tests (RDT) [11]. Treatment is based on the use of artemether/lumefantrine for *P*. *falciparum* and chloroquine + primaquine for *P*. *vivax*, according to the respective parasite sensitivity to these anti-malarials. Prevention activities cover a wide variety of strategies, ranging from preventive health education to vector control measures, including the use of insecticide-impregnated nets (ITNs) and in-door residual spraying (IRS) with long-lasting insecticides, particularly DDT, deltametrin and lambda-cyhalotrin. Monitoring includes passive case detection performed by a network of community collaborators that contribute with diagnosis, monitoring and evaluation of the NMCP, whereas active detection of malaria cases is only performed during epidemic outbreaks [9,10].

Besides the usual activities of the NMCP, a project sponsored by the Global Fund (GF) for AIDS, tuberculosis and malaria between 2005 and 2010 was directed at reducing malaria transmission at adjacent country borders (PAMAFRO) that included Peru, Ecuador, Colombia, and Venezuela. This project significantly contributed to a decline in malaria transmission, particularly in bordering departments such as Nariño in the south-western region of Colombia. Moreover, since 2010, a new five-year GF project is being conducted in the five departments (=states) that contribute >80% of malaria cases in Colombia: Cordoba, Antioquia, Chocó, Valle del Cauca, and Cauca. The strategy of this ongoing GF project is to train community workers who will be responsible for leading disease prevention processes: organization of surveillance committees, ensuring notification and promoting access to RDT-based diagnosis and immediate treatment.

In spite of the significant national efforts against malaria, the impact of all these activities has been hampered by situations such as armed conflicts, illegal agriculture and mining, social inequality, climate change, and the rather cumbersome administrative structure of the NMCP [12]. The latter includes decentralization of the NMCP, which has been attempting to transfer control activities to the social security system (SSS) since 2000. The SSS is composed of health provider enterprises (EPS) and health provider institutions (IPS) with relatively low malaria technical and operational capacity in some departments and municipalities, together with a general lack of political will and social responsibility.

Because of the paucity of reports that describe the current national malaria situation, the aim of this study was to describe the characteristics of malaria cases reported in Colombia during 2010 as a starting point for prioritizing a prevention and control agenda.

## Methods

### Study design and case definitions

A descriptive study was conducted using data from SIVIGILA 2010. Definitions established by this surveillance system were adopted for laboratory-confirmed uncomplicated malaria cases, probable cases, and severe and complicated malaria [8], as described in Table 1.

**Table 1:** Definitions used in this study*

**Complication**	**Description**
**Cerebral malaria**	Altered state of consciousness (irreversible coma), unconsciousness with the possibility of waking up, prostration, extreme weakness (patient cannot sit or stand), generalized convulsions or behavioral disturbance.
**Renal complication**	Serum creatinine > 3.0 mg/dl and/or urine vol < 400 ml in 24 hours (adults) or <12 ml/kg of body weight in 24 hours (children)
**Lung or respiratory distress syndrome**	Increased respiratory rate at admission, presence of abnormal lung sounds, such as wheezing, rhonchi, rales, and radiographic changes consistent with pulmonary oedema.
**Shock**	Systolic blood pressure <70 mm Hg in adults or <50 mm Hg in children.
**Hypoglycaemia**	Glucose < 40 mg/dl.
**Hyperemesis**	Uncontrollable and frequent vomiting, >5 times in 24 hours, which prevents anti-malarial treatment orally.
**Hyperpyrexia**	Axillary temperature >39.5°C
**Severe anemia**	Haematocrit < 15% or Hb <5 g/dl, Spontaneous bleeding or disseminated intravascular coagulation (DIC), acidaemia/acidosis (clinical signs), macroscopic haemoglobinuria
**Complicated malaria case**	Any patient with probable case of malaria complicated by the presence of asexual (trophozoites/schizonts) of *P*. *falciparum* confirmed by parasitological examination and who has been ruled out another etiology, or any patient with confirmed cases of malaria with >50,000 parasite asexual forms /μL (hyperparasitaemia)
**Death from malaria**	Patient with signs and symptoms of complicated malaria, with confirmed diagnose of *P*. *falciparum* (or *P*. *vivax*) or associated infection.
**Complication hepatic jaundice**	Total serum bilirubin >3 mg/dl and altered liver function tests.

* SIVIGILA definitions.

### Descriptive variables

Variables considered in the descriptive analysis were: parasite species, number of uncomplicated malaria and lethal cases during the year, department and municipality of origin, duration (months) of consultation, age, gender, type of health regime, and ethnicity. Additionally, “time between symptoms and consultation” was included as a variable corresponding to the time between the symptoms onset and the date of consultation.

### Database mining

Databases were refined taking into account the recommendations of the monitoring system. In general, these recommendations address validation rules that take into account the variable code, date of service, epidemiological week, type identification, identification number, primary data generating units (PDGU) and information units (IU), which emphasize the assessment of repeated and duplicate cases [13].

### Statistical methods

Information was processed using spreadsheets and Microsoft Excel for analysis, using Tableau statistical package, version 6.0. Univariate analyses were performed for all variables included. The proportions, *vivax*/*falciparum* ratio, and measures of central tendency and dispersion were calculated. The API was calculated by relating the total number of malaria cases (117,108) in 2010 with the population at mid-term risk for the same year (10.2 million), multiplied by a thousand. The chi-square test was used to assess differences between the variables reported in 2010 compared with the previous year, and to calculate P values; which was considered statistically significant when <0.05.

## Results

**Figure 1:**
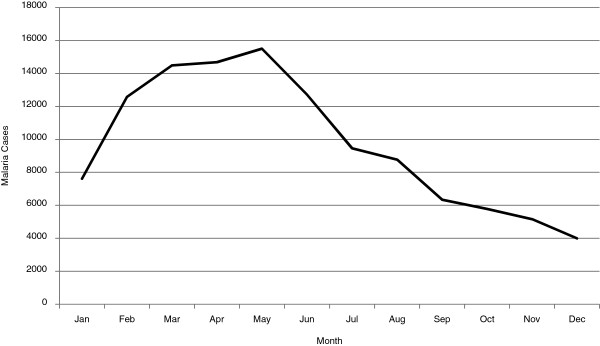
Distribution of malaria cases by month, Colombia, 2010.

A total of 117,108 diagnosed malaria cases were recorded during 2010 by SIVIGILA; 77,586 of which, equivalent to 66.3% of all cases for the year, were reported during the first half of the year. These numbers corresponded to a 32.6% increase from the 78,498 cases reported in 2009 (p < 0.001), which constituted an epidemic year (Figure 1).

**Figure 2:**
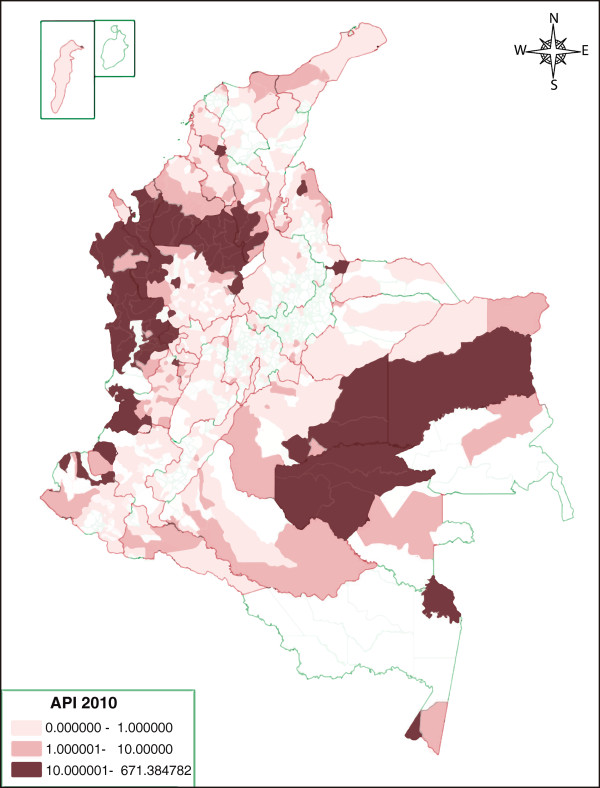
Annual parasite index by territorial entity.

Twenty percent (7/32) of the Colombian departments, represented by the departments of Antioquia, Cordoba, Chocó, Guaviare, Vichada, Valle del Cauca and Amazon, presented an IPA above the national average of 10.5 cases/1,000 habitants (Figure 2). Additionally, stratification by territorial entity (departments and districts) according to the IPA indicated that in these seven states, 27.7% of the rural population presented 87.9% of cases, leading to an IPA >10/1,000, and representing areas of high malaria transmission risk. As shown in Table 2, most cases were reported in Antioquia (39.7%), followed by Chocó (19.2%), Córdoba (17.2%), Valle (5.4%), Guaviare (5.0%), Nariño (3.5%), and Bolivar (3.1%) cases. Another 14 departments accounting for 33.2% of the rural population recorded 11.0% of cases and, therefore, displayed an IPA between 1-9/1,000, and were classified as medium-risk regions. In the remaining 15 entities, with 39.1% of the rural population, reported only 0.9% of the cases, presented an IPA <1.0/1,000, and were, therefore, considered of low risk. Table 2 shows the specific increase in the number of malaria cases observed in the period 2009-2010 by territorial entity. Despite the fact that not all of the territorial entities reported an increase in malaria, five of the most endemic (Antioquia, Chocó, Cordoba, Guaviare and Valle) reported an average increase of 44%.

**Table 2:** Distribution of malaria cases by parasite species and department (=state)

**Territorial entity**	**2009**				**2009**				
	***P*.*vivax***		***P*.*falciparum***		***P*.*vivax***		***P*.*falciparum***		**Rate of change**
	**n**	**%**	**n**	**%**	**n**	**%**	**n**	**%**	**%**
Antioquia	26.341	33,42	6.059	7,69	36.620	31,27	9.463	8,08	29,7
Córdoba	10.248	13	3.008	3,82	14.281	12,19	5.696	4,86	33,6
Chocó	5.180	6,57	2.931	3,72	12.543	10,71	9.608	8,2	63,4
Guaviare	1.065	4,3	1.184	1,5	4.940	4,22	877	0,75	21,4
Nariño	3.388	1,35	4.505	5,72	693	0,59	3.381	2,89	-36,7
Valle del Cauca	991	1,26	913	1,16	5.061	4,32	1.213	1,04	69,7
Meta	1.427	1,81	265	0,34	1.069	0,91	372	0,32	-17,4
Cauca	149	0,19	1.730	2,19	151	0,13	708	0,6	-118,7
Amazonas	1.729	2,19	82	0,1	747	0,64	33	0,03	-132,2
Vichada	1.226	1,56	246	0,31	535	0,46	269	0,23	-83,1
Risaralda	1.010	1,28	29	0,04	1.053	0,9	139	0,12	12,8
Bolívar	849	1,08	65	0,08	2,6	2,22	755	0,64	-20,6
La Guajira	955	1,21	24	0,03	315	0,27	42	0,04	-174,2
Putumayo	721	0,91	6	0,01	236	0,2	11	0,01	-194,3
Norte Santander	237	0,3	1	0	346	0,3	2	0	31,6
Caquetá	169	0,21	44	0,06	220	0,19	33	0,03	15,8
Sucre	182	0,23	27	0,03	177	0,15	23	0,02	-4,5
Vaupés	100	0,13	24	0,03	205	0,18	4	0	40,7
Caldas	26	0,03	28	0,04	201	0,17	21	0,02	75,7
Magdalena	104	0,13	61	0,08	35	0,03	7	0,01	-292,9
Guainía	105	0,13	14	0,02	72	0,06	4	0	-56,6
Quindío	76	0,1	11	0,01	83	0,07	22	0,02	17,1
Boyacá	62	0,08	4	0,01	118	0,1	5	0	46,3
Outside	102	0,13	7	0,01	55	0,05	10	0,01	-67,7
Cundinamarca	40	0,05	4	0,01	97	0,08	8	0,01	58,1
Tolima	55	0,07	9	0,01	67	0,06	11	0,01	17,9
Santander	53	0,07	16	0,02	61	0,05	8	0,01	0,0
Without information	42	0,05	16	0,02	50	0,04	13	0,01	7,9
Cesar	24	0,03	3	0	65	0,06	2	0	59,7
Casanare	33	0,04	10	0,01	36	0,03	7	0,01	0,0
Huila	22	0,03	4	0,01	22	0,02	3	0	-4,0
Arauca	30	0,04	3	0	13	0,01		0	-153,8
Atlántico	23	0,03	6	0,01	10	0,01	1	0	-163,6
Santa Marta		0		0	38	0,03	2	0	100,0
Cartagena		0		0	16	0,01	12	0,01	100,0
Bogotá	16	0,02		0	5	0	5	0	-60,0
Barranquilla		0		0	15	0,01	4	0	100,0
San Andrés		0	1	0	5	0	3	0	87,5
Total	56.780	72,04	21.340	21.340	82.856	70,75	32.777	27,99	

Colombia, 2009 – 2010.

Source: SIVIGILA.

**Figure 3:**
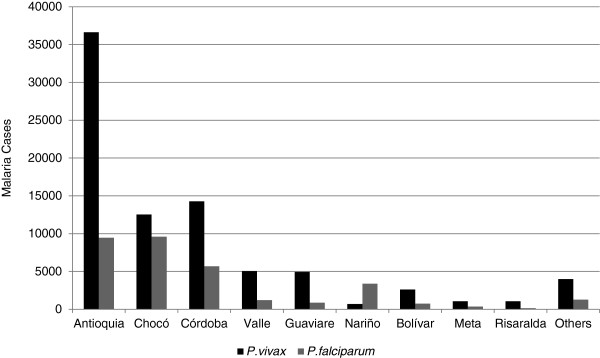
Distribution of malaria by department, Colombia, 2010.

**Figure 4:**
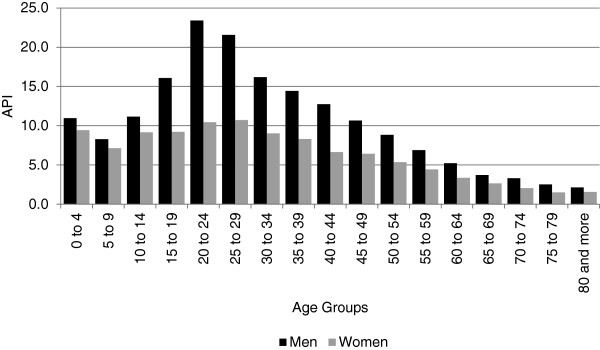
Annual parasite index by gender and age, Colombia, 2010.

Most cases were caused by only one parasite species. *Plasmodium vivax* was identified in 82,856 (70.8%) cases, *P*. *falciparum* in 32,777 (28.0%) cases, and *P*. *malariae* in 47 (0.04%) cases. Additionally, 1,428 cases of mixed infections (*P*. *falciparum* and *P*. *vivax*, 1.2%) were reported. The ratio of *P*. *vivax* to *P*. *falciparum* cases was 2.5 to 1. Most of the territorial entities had a higher proportion of *P*. *vivax*, except in Nariño, where *P*. *falciparum* was the predominant species (*P*. *vivax*/ *P*. *falciparum* = 1:5) (Figure 3). Likewise, most cases (75,636 = 64.6%) occurred in men and 41,472 = 35.4% in women. Age ranged from the first day of life to 101 years (median age, 22 years). The IPA distribution by sex and age was higher for men, with similar distribution for all age groups (Figure 4). The higher API values converged on the group of 20 to 24 years: 72.9% in men and 27.1% women.

Of people affected by malaria, 73,805 (63.0%) were from rural areas; 24,779 (21.2%) from small villages and 18,508 (15.8%) from municipalities. Regarding the social security system, nearly two-thirds of the participants were enrolled (68,029, 58.1%), while 49,070 (41.9%) cases were not. The ethnic group analysis showed that 27,382 (23.4%) occurred in groups of African descent; 9,311 (8.0%) in Native Americans; 299 (0.3%) in *raizales*; 98 (0.1%) in Gypsy; 22 (0.02%) in *palenqueros*; and 79,988 (68.3%) in white/*mestizo* population. In terms of occupation, 75.4% of the reported cases were categorized as follows: 29,294 (25.0%) students; 16,769 (14.3%) miners; 15,072 (12.9%) housewives; 10,264 (8.8%) children; 8,077 (6.9%) farmers; 5,329 (4.6%) soldiers; 3,494 (3.0%) forest workers. In relation to the time between the symptoms onset and the time of diagnosis, there was a median of two days (range: 0-309). 54.2% of the cases consulted within 48 hours after symptoms onset (Table 3). Eight departments had clinical care percentages that exceeded the national average, including Antioquia and Cordoba, which qualified as high risk according to the API with 57.0 and 64.5%, respectively.

**Table 3:** Time between onset of symptoms and time of consultation

**Time**	**P. vivax**		**P. falciparum**		**Mixed infections**		**P. malariae**		**Total**	
	**n**	**%**	**n**	**%**	**n**	**%**	**n**	**%**	**N**	**%**
24 hours	29.276	35,3	11.904	36,3	388	27,2	19	40,4	41.587	35,5
48 hours	15.612	18,8	6.043	18,4	278	19,5	5	10,6	21.938	18,7
72 hours	13.213	15,9	4.995	15,2	226	15,8	8	17,0	18.442	15,7
More than 96 hours	24.753	29,9	9.828	30,0	536	37,5	15	31,9	35.132	30,0
Don’t know	2	0,0	7	0,0		0,0		0,0	9	0,0
Total	82.856	100,0	32.777	100,0	1.428	100,0	47	100,0	117.108	100,0

Colombia, 2010.

Source: SIVIGILA.

### Malaria in children under 15 years of age

Of the 117,108 cases reported in 2010, 35,458 (30.3%) occurred in children under 15 years; 840 (2.4%) were aged under one year; 11,917 (33.6%) belonged to the group of one to four years; 9,838 (27.7%) were in the group of five to nine years; and 12,863 (36.3%) were in the ten to 14 year-age group. Parasite species distribution in this group was 26,142 (73.7%) with *P*. *vivax* and 8,998 (25.4%) with *P*. *falciparum*. The proportion of *P*. *vivax* cases in the group ≤15 years was 1.25 times higher than in ≥15 (95% CI = 1.21-1.29, p = 0.000). Malaria cases in children <15 years were concentrated in three territorial entities, 67,292 (82.5%) with an API ≥10: Antioquia: 12,204 (34.4%); Chocó: 9,033 (25.5%); Cordoba: 8,030 (22.6%). About half of the cases (54.7%) in this age group consulted within the first 48 hours after symptom onset.

### Malaria during pregnancy

A total of 29,345 malaria cases occurred in women of reproductive age (ten to 54 years); 251 (0.9%) of which were pregnant, with >70% of gestational malaria cases concentrated in a few departments. Antioquia reported 45.8% of the cases (115/251; p = 0.016); followed by Córdoba: 11.6% (29/251; p = 0.005); Choco: 8.4% (21/251; p = 0.01); Guaviare: 7.6% (19/251; p = 0.011). Almost half of the cases (45.8%) consulted within the first 48 hours after symptom onset and no significant difference was observed in the parasite species presented by pregnant and non-pregnant women.

### Complicated malaria

A total of 623 (0.5%) complicated malaria cases were reported, caused almost equally by *P*. *vivax* and *P*. *falciparum* with most patients (70.1%) in the age group of 15 to 64 years, and the remainder (27.9%) occurred in children <15 years old. Liver failure (39.2%) and renal failure (26.8%) were the most frequently reported complications (Table 4).

**Table 4:** Complicated malaria cases by parasite species

	**Parasite species**					
	***P*.*vivax*(n = 293)**	***P*.*falciparum*(n = 282)**	**mixed (n = 32)**	***P*.*malariae*(n = 6)**	**unreported (n = 10)**	**Total (n = 623)**
Cerebral	44 (7,1%)	59 (9,5%)	6 (1,0%)	1 (0,2%)	0	110 (17,7%)
Renal	66 (10,6%)	88 (14,1%)	10 (1,6%)	2 (0,3%)	1 (0,2%)	167 (26,8%)
Liver	130 (20,9%)	99 (15,9%)	14 (2,2%)	1 (0,2%)	0	244 (39,2%)
Pulmonary	34 (5,5%)	31 (5,0%)	1 (0,2%)	2 (0,3%)	0	68 (10,9%)
unreported	19 (3,0%)	5 (0,8%)	1 (0,2%)	0	9 (1,4%)	34 (5,5%)

Source: SIVIGILA.

### Deaths from malaria

Twenty-three (0.02%) deaths attributable to malaria were produced in equal proportion by *P*. *vivax* (47.8%) and *P*. *falciparum* (47.8%), with most cases (69.6%) occurring in patients aged 0 to 29 years, although the range varied between ten months to 80 years old (median age, 27 years) These deaths occurred in 15 (65.2%) women and 8 (34.8%) men. The most frequent complications were brain (47.8%) and lung (30.4%) diseases, and surprisingly, 43.5% of the cases were recorded as having consulted within the first 48 hours after symptom onset. These deaths occurred in non-pregnant women (15) and men (eight) from Chocó (seven); half in Antioquia, Córdoba, Valle del Cauca and Risaralda departments which reported three deaths each. In about half of the fatal cases (47.8%), self-medication was associated to the case reported.

## Discussion

Although a decreasing trend in the number of annual malaria cases has been reported in Colombia since 2001, transmission peaks were observed in 2007 and 2010, the latter representing a 32.6% increase from the previous year (2009). Such an increase corresponds to a seasonal epidemic transmission outbreak that coincided with the *para*-*quinquenal* malaria transmission peak period described in areas of low transmission [14]. This outbreak was more evident in the traditionally highest transmission regions, i.e., departments of Antioquia, Córdoba, Chocó, Valle, Guaviare, Nariño, and Bolivar. Although this cyclical behaviour is considered to be due mainly to climatic changes induced by the El Niño phenomenon, several other determinants may have contributed to the malaria transmission increase. Social factors such as increased population migration through high malaria transmission areas occurred during the same period due to illegal agriculture and mining, which both resulted in environmental degradation. In addition, significant community displacement was forced by the growing presence of both legal military corps and insurgent troops through areas of high malaria transmission.

Because of its geographical location, most regions of Colombia present interposed rainy and dry seasons during the entire year, leading to relatively constant malaria transmission without the classical epidemic peaks observed in other latitudes [15,16]. It is not clear why most cases (66.3%) presented during the first half of the year during which the most endemic regions presented a dry behaviour associated with El Niño, followed during the second half by La Niña (rainy season). This could have been due in part to inadequate administrative infrastructure at NMCP, as well as to the political instability that was most evident in endemic regions. First, it appears like there were deficiencies in providing timely diagnosis and treatment due to administrative issues that led to insufficient recruitment and hiring of microscopists in rural areas. Second, the staffing constraints in NMCP appeared to be further complicated by limitations placed on routine performance of prevention and control activities due to security concerns arising from the intensification of social conflict in the most endemic areas [7]. Indeed, the most endemic departments, Antioquia, Córdoba, Chocó, Valle, Guaviare, Nariño and Bolivar, presented IPA above the national mean, 10.5 cases/1,000 inhabitants; and all are known to be affected by the conditions described above. In these regions, mining and illegal agriculture usually leads to environment damage, and establishment of temporary settlements with poor households. Third, although records indicate that two-thirds of cases were reported in individuals who in theory had social security (EPS, IPS), in reality, under the social conditions described, access to health care is not adequate when these communities are left in the hands of a debilitated NMCP.

Antioquia and Cordoba corresponded to the highest risk according to the API with 57.0 and 64.5%, respectively, and together with Chocó, accounted for the greatest percentage (82.5%) of cases in children <15 years of age, which correlates with high malaria transmission in these areas [17]. The parasite species distribution did not present any change in this epidemic year as compared with the proportion over the last decade where most cases (~74%) were produced by *P*. *vivax*. This higher prevalence of *P*. *vivax* was even greater in the group ≤15 years old (1.25 times) than in older individuals. As expected, the most endemic departments presented a greater number of cases in children and in pregnant women.

It is interesting to note that in spite of the epidemic behaviour during this period, and that most cases (63.0%) were from rural areas where there is inadequate access to diagnosis and treatment, over half of the malaria cases consulted early after symptoms onset (first 48 hours). This early consultation may explain the low frequency of complicated (0.5%) and lethal (0.02%) cases. In addition, the high prevalence of acute but clinically benign cases may also be due to the fact that most cases (70.8%) were caused by *P*. *vivax* with only 1.2% of reported cases corresponding to mixed infections. The low frequency of complications and mortality may also be due to the population displacements that occurred within endemic regions and that probably involved communities that had already been significantly exposed to malaria.

It is of concern that about one-third of the cases (37%) were reported as originating in urban and peri-urban settings. It is extremely important to determine if there were actually 15% of urban cases or if these corresponded to inaccurately recorded cases. Despite the great risk of maintaining urban malaria transmission, little is known about the potential vector species in such localities. A total of 21 municipalities with well-defined urban areas were reported as having such cases. Even if the cases are occurring in less formally urbanized areas, increased attention must be devoted to elucidate the origin of these cases, as well as on intervening in such settings, which would not only decrease the risk of transmission, but would also prove to be more cost effective. Although, transmission may be occurring in urban areas recently colonized, where mosquito vectors are becoming adapted [18,19], it might also be that patients report false addresses or case origin, just to avoid being associated to illegal activities. In any case, a high proportion of the urban population lives in extreme poverty, with limited access to health and education [20] and have higher risk of severe disease due to the lack of protective immunity.

Although most cases (64.6%) occurred in men with higher API values converging in the group 20 to 29 years of age, one third (30.3%) occurred in children under 15 years old. In contrast to holo-endemic and hyper-endemic regions in Africa and Asia (PNG) where greater morbidity and mortality occur in children and infants, only a limited proportion of the cases presented in children <5 years of age. In this case, one-third of the cases occurred in children <15 years/age, which may indicate transmission in the household and school neighborhood. Fewer malaria cases were reported in women, although ~26% of these occurred in women of reproductive age (ten to 54 years), <1% of them were pregnant at the time of diagnosis. Previous studies conducted in areas of high risk for the disease had indicated a higher number of malaria cases during pregnancy in Colombia with prevalences between nine and 10.4% [21,22]. The contrast of these figures to the ones reported here may be due to the fact that those studies were performed in areas of highest transmission, whereas here we have included surveillance data of the entire country. Indeed > 65% of the cases were reported from Antioquia (45.8%); Córdoba (11.6%); and Chocó (8.4%).

Another possible explanation for these contrasting figures is an inaccurate or incomplete recording of the gestational status at the time of malaria diagnosis, where information on the pregnancy status of malarial women of child-bearing age might have not been properly recorded resulting in a reduction in the number of reported malaria cases in pregnant women. This has been observed in Brazil and other countries of the region, where the actual frequency of malaria and pregnancy is unknown [23]. Additionally, studies focused on malaria and pregnancy, have focused more attention to properly recording the study data. In a previous patient capture - recapture study conducted in Urabá, of estimated a that SIVIGILA presented underreporting of 80% of pregnant women with malaria [24]. This stresses the need of better training for staff performing data collection by implementing regular courses, strengthening awareness and reporting procedures.

Neither the malaria distribution by ethnic group nor the distribution by occupation appears to have changed from that reported in 2009. Almost 70% of cases occurred in white and *mestizo* populations, followed by ~23% reported in African descent groups. Likewise, students, miners, housewives, and children of both genders accounted for ~60% of the cases. It is not surprising that only 623 (~0.5%) complicated malaria cases were reported since early diagnosis was a rule, this consequently explains the low rate of mortality. Although complicated cases were distributed in all age groups, lethal cases occurred in young patients (<29 years) although not necessarily in children. It is not surprising that these complicated cases were equally distributed between the two parasite species as *P*. *falciparum* was less frequent but is more pathogenic than *P*. *vivax*. In contrast, *P*. *vivax*, which have been considered benign, has been recently reported to be responsible for severe and complicated cases as well as for significant mortality [25]. The incidence of clinical complications by *P*. *vivax* is unknown and the clinical profile of this species has been poorly studied and is probably not well recognized by physicians. Surprisingly, >40% of the complicated cases were recorded as having consulted within the first 48 hours after symptom onset. Data analysed here correspond to malaria cases diagnosed by thick smear, however, as PCR malaria diagnose has been introduced, recent studies have started reporting that malaria by *P*. *vivax* could be as deadly as malaria by *P*. *falciparum*[26]. Although some hypotheses suggest that the chloroquine-primaquine combination, which has been the first-line of treatment for *P*. *vivax* for almost 50 years, may not be equally effective nowadays, there is no evidence for this decrease in effectiveness in Colombia. Similarly, other studies report wrong diagnosis of the species [27] which affects the clinical decision about the recommend treatment [28]. In fact, individuals experimentally infected in the context of vaccine clinical trials have responded to this combination, with parasite clearance between 24-48 hours after treatment initiation [29,30]. One could speculate that there was poor compliance with the official anti-malarial therapy, particularly in those fatal cases where self-medication was reported.

This paper presents constraints arising from taking information from a secondary source with possible notification mistakes, presence of ignored or uncompleted fields, and lack of consistency in some variables. There may also be errors in the digitization of information that could lead to underestimating the real number of malaria cases [31]. In addition, there may be misclassification of cases given by errors in diagnosis and lack of adherence to case definitions used for surveillance. The lack of special units for analysis of complicated malaria or malaria deaths at the NMCP and the reluctance of the SSS to assume the responsibility of malaria diagnoses and treatment are currently limiting the quality of information. Currently, the Ministry of Health is articulating different information systems and ongoing studies are being complemented with information from other sources, such as from individual records services (known as RIPS = Registros Individuales de Prestación de Servicios de Salud), as well as from mortality records and from entomological surveillance data. Quantitative and descriptive studies could help to better establish the real magnitude of the events and analytical studies could determine other factors associated with the presence of the disease.

During the last two years (2011-2012), there has been a significant malaria decrease (~61,000 cases/year), despite the fact that major determinants, such as population migration, illegal agriculture and mining appears to be growing as well as the presence of both legal military corps and insurgent troops. This indicates the great efforts of the country to decrease malaria transmission, during the last couple of years a more timely diagnosis and treatment as well as prevention and control activities are being deployed jointly between the NMCP and the GF project, which appear to be producing a beneficial impact.

These local factors may explain why whereas countries like those of Central America or Peru and Ecuador are substantially reducing malaria transmission, Colombia remains with significantly greater transmission. However the evolution during the last decade has brought transmission from ~200,000 cases/year [32], to ~61,000 cases in 2012 (SIVIGILA 2013), and currently, greater efforts are being invested to maintain the decreasing trend, although permanent monitoring to avoid epidemic outbreaks is still required i.e. by prioritizing critical control activities such as effective vector management and educational activities as well as greater efforts to overcome administrative hurdles that limit a more robust diagnosis and treatment system. In the context of the GF project, the COMBI strategy (social mobilization for behaviour change) is successfully leading to community participation in prevention and control activities that therefore become more efficient and cost-effective [20,33-35]. Moreover, in the framework of national and international research projects such as Malaria Research Network sponsored by the Colombian government, and (International Centers of Excellence for Malaria Research–ICEMR) sponsored by the US National Institutes of Health (NIH) the NMCP has been involved in translation malaria research projects, including studies leading to define the etiology and potential co-morbidities of complicated and lethal *P*. *vivax* cases as well as the real intensity of urban malaria transmission. These new initiatives are likely to generate new strategies to force the malaria incidence downwards in the following years.

## Competing interests

The authors declare that they have no competing interests.

## Authors’ contributions

JP and PC conceived and designed the study. SH, AV and PC wrote the manuscript. All authors read and approved the final manuscript.
